# Antigen-Based Immune Therapeutics for Type 1 Diabetes: Magic Bullets or Ordinary Blanks?

**DOI:** 10.1155/2011/286248

**Published:** 2011-05-14

**Authors:** Slobodan Culina, Christian Boitard, Roberto Mallone

**Affiliations:** ^1^INSERM, U986, DeAR Lab Avenir, Saint Vincent de Paul Hospital, 82 avenue Denfert Rochereau, 75674 Paris Cedex 14, France; ^2^Université Paris-Descartes, 75006 Paris, France; ^3^Assistance Publique Hôpitaux de Paris, Hôtel Dieu, Service de Diabétologie, 75181 Paris, France

## Abstract

The ideal drug of modern medicine is the one that achieves its therapeutic target with minimal adverse effects. Immune therapy of Type 1 diabetes (T1D) is no exception, and knowledge of the antigens targeted by pathogenic T cells offers a unique opportunity towards this goal. Different antigen formulations are being considered, such as proteins or peptides, either in their native form or modified *ad hoc*, DNA plasmids, and cell-based agents. Translation from mouse to human should take into account important differences, particularly in the time scale of autoimmune progression, and intervention. Critical parameters such as administration route, dosing and interval remain largely empirical and need to be further dissected. T1D staging through immune surrogate markers before and after treatment will be key in understanding therapeutic actions and to finally turn ordinary blanks into magic bullets.

## 1. Introduction

Type 1 diabetes (T1D), one of the most common autoimmune diseases, stems from defects in central and peripheral tolerance that lead to progressive T-cell-mediated destruction of insulin-producing *β* cells in pancreatic islets. Clinically, this destruction results in the inability of affected individuals to produce the insulin required to properly regulate glucose metabolism, causing substantial morbidity and mortality. Although metabolic derangements are only the consequences of the underlying autoimmune pathogenesis, they remain the only targets of mainstream insulin therapy. Immune-targeted interventions which could correct autoimmune mechanisms are, therefore, intensively sought as a more rational approach. Preclinical studies largely take advantage of the nonobese diabetic (NOD) mouse, which is one rare instance of autoimmune model, where disease develops spontaneously rather than being induced by experimental manipulation.

## 2. Translating the NOD Mouse into Human

There is a long list of immune biologics that have spectacular effects in preventing T1D in the NOD mouse. However, only a handful of them has shown some effect once translated into clinical trials. There are a number of reasons explaining these discrepancies which should be kept in mind. First is the phylogenetic difference between the murine and human immune system, which is not surprising considering that the two species diverged ~70 million years ago [1]. Second, although the NOD is a spontaneous model of T1D which is linked to MHC susceptibility haplotypes and involves a complex immune interplay like in human, there are important discrepancies. These include characteristics of the insulitis infiltrate, autoantibody (aAb) specificities, and association with other autoimmune manifestations (e.g., sialitis). Third, the NOD is an inbred strain composed of genetically identical animals. As such, it can be assimilated to one single T1D patient, and indeed a very peculiar one. Further underrepresenting human disease, these mice are kept under identical environmental conditions, protected from most infectious threats. Fourth, disease and treatment kinetics are quite different, as NOD mice are most commonly treated for preventing diabetes at an early stage, before the appearance of any circulating autoimmune marker such as anti-insulin aAbs (IAA). This is rarely possible in humans, as further discussed. Indeed, when we look at immune therapies which work in the NOD mouse once the disease has become clinically overt, the list of effective treatments falls much shorter. The challenge that intervention trials face in humans is thus a formidable one. For this reason, we will focus our discussion on antigen- (Ag-) specific agents that have already been tested or are soon to enter clinical trials, reasoning that our goal is to cure men rather than mice. 

## 3. Immune Therapy for Type 1 Diabetes: Whom for?

Studies in the NOD mouse suggest that the great majority of the *β*-cell mass (~75%) has already been destroyed by the time of diabetes onset [2]. A recent meta-analysis and mathematical modeling of three landmark histopathological studies of human T1D pancreata [3–5] suggests that this is also the case in humans [6]. However, the extent of *β*-cell destruction varies with age, ranging from 85% in children to 40% in adults [6]. This is probably due to a combination of factors, which include a physiological age-related decline in *β*-cell mass, differences in insulin needs according to body weight, growth and insulin sensitivity, and the degree of *β*-cell autoimmunity, which may be more aggressive in younger patients. One limitation of most histopathological studies is that measurement of residual *β*-cell mass was based on enumeration of insulin-positive cells within the islets. It is, thus, possible that viable *β* cells not producing insulin because of functional impairment are missed, leading to an overestimation. It is also difficult to relate these histopathological estimates of residual *β*-cell mass with those of residual *β*-cell function, as determined by stimulated C-peptide secretion. However, recent studies suggest that insulin production may be more substantial at diagnosis than had been previously appreciated and that residual insulin secretion may persist in a subgroup of T1D patients [7]. Further complicating the picture, the question of whether significant *β*-cell renewal occurs in T1D patients remains unsettled [8]. 

**Figure 1:**
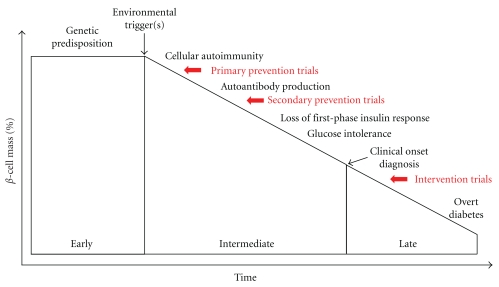
Stages of disease progression and intervention in T1D. Progression over time (*X*-axis) from simple genetic susceptibility to *β*-cell autoimmunity and T1D is plotted against residual *β*-cell mass (*Y*-axis). The time points at which immune therapies are administered are shown in red.

 Whichever the real extent of *β*-cell destruction at the time of T1D onset, it is clear that interventions aimed at correcting immune mechanisms should be implemented as early as possible, ideally at a preclinical stage in as yet healthy subjects at risk of developing disease (Figure 1). A related problem is, therefore, to reliably identify these subjects. Although genetic markers (particularly HLA Class II susceptibility alleles) and serum aAbs against the *β*-cell Ags insulin, glutamic acid decarboxylase (GAD), insulinoma-associated protein 2 (IA-2), and zinc transporter 8 (ZnT8) greatly help to stratify T1D risk, they fall short in accuracy, as they can predict the risk but not the time course of disease development (i.e., the “if”, but not the “when”), and much less when only one aAb marker is present. The accuracy of these predictions needs to be very stringent if used to decide enrollment into a clinical trial, because the risk-benefit balance is a delicate one for T1D. We are not facing a rapidly lethal disease but rather one that, despite difficult daily management and long-term complications, carries a life expectancy which is getting closer and closer to normal. This benign prognosis needs to be weighed against the risks of experimental immune therapeutics, whose long-term adverse events are frequently unknown. It is, therefore, ethically acceptable to trial only those subjects in whom T1D will eventually develop. Given the efforts and difficulties needed to recruit an adequate number of at-risk subjects, T1D prevention studies have been quite limited and performed mostly in subjects at very high risk. Trials in newly diagnosed T1D patients are more common, with the inner constraint that, even if the autoimmune process is effectively halted, there is limited clinical benefit to be expected from rescuing the residual *β*-cell mass. This is particularly daunting in T1D children, in whom the remaining *β* cells are fewer than in adults and may be as few as 15% [6]. In this respect, combination of immune biologics with strategies aimed at replacing or regenerating lost *β* cells may expand the optimal time window for intervention. 

## 4. Immune Suppression versus Tolerance Restoration

Two broad strategies could theoretically be followed to intervene on *β*-cell autoimmunity. The first one would consist of correcting the environmental causes triggering autoimmunity. However, this is certainly the most frustrating failure of half a century of T1D research, which has elucidated a wealth of immune mechanisms without identifying the environmental *primi moventes*. The only emerging exception may be the role of the gut microbiome, as data obtained in the NOD mouse suggests that altering composition of intestinal flora may offer new treatment paths in the future [9]. Factors responsible for insulin resistance are also emerging contributors to T1D development, but they probably play a precipitating rather than causative role. Nonetheless, insulin resistance has recently been recognized as a powerful T1D risk factor [10], and clinical trials with drugs such as metformin acting on metabolic pathways keep being investigated [11–13].

The second, currently more viable, strategy is to correct immune mechanisms. In doing this, an important difference needs to be made between immune suppression and tolerance restoration. With immune suppression, a generalized status of lessened reactivity is induced so that the body has decreased responses to Ag challenges of any kind. This exposes to increased risks of infection and secondary tumor growth. Moreover, treatment needs to be lifelong, as its effects are lost after discontinuation. Immune suppression is typically induced by drugs that act on common signaling pathways used by key immune cells. Examples of such drugs used in the past are cyclosporin A [14] and, in more recent years, anti-CD25 monoclonal antibodies (mAbs) and mycofenolate mofetil, used either alone or in combination [15]. 

 With immune tolerance restoration, the induced effect is not generalized but limited to specific types of responses, ideally only to responses specific to *β*-cell Ags. Thus, the immune system remains capable of responding to infectious and tumoral threats. Treatment should be limited in time, while the effects should persist after discontinuation. This is because an “active” effect is induced by boosting natural immune tolerance mechanisms. With this frame of reference, it is evident why immune restoration strategies are preferable. Given their more selective action and limited duration of treatment, their risk-benefit balance is more attractive, especially if they have to be applied in prevention trials. Although several non-Ag-specific therapeutic mAbs are also under scrutiny [16], agents that exploit the target *β*-cell Ags themselves may offer the best warranties of Ag-specific immune tolerance.

## 5. Which *β*-Cell Antigens?

The list of *β*-cell Ags relevant to T1D autoimmunity is quite long [17], but few of them have resisted the proof of time and proved to be key targets of aAb and/or T-cell responses. These Ags are insulin and its precursors proinsulin (PI) and preproinsulin (PPI), GAD, IA-2, ZnT8, and islet glucose-6-phosphatase catalytic subunit-related protein (IGRP). The latter is the only one to be exclusively described as a target of T cells [18, 19], as aAbs have been sought after but not yet found.

A popular tenet in autoimmunity is that there could be one primary self Ag which initiates pathogenesis. Tissue destruction through targeting of this Ag could further release other ones, thus amplifying the autoimmune cascade through a phenomenon known as epitope spreading. In the NOD mouse, insulin has been identified as the initiating *β*-cell Ag. The importance of insulin is supported by data on insulin knockout NOD mice. Different from humans, rodents express two isoforms, referred to as insulin 1 and 2. NOD mice defective for the insulin 2 gene, the prevalent isoform in the thymus, display accelerated T1D [20], likely related to defective deletion of insulin-reactive T cells [21]. Conversely, NOD mice defective for the insulin 1 gene, one of the two isoforms expressed in the islets, are less susceptible to T1D [22]. However, insulin 1 knockout islets transplanted into recently diabetic wild-type NOD mice become infiltrated and only transiently reverse T1D, suggesting that insulin is an early but not exclusive target [22]. More recent evidence from Nakayama et al. further suggests that insulin may be the initiating *β*-cell Ag in T1D [23]. These authors produced NOD mice, where the endogenous insulin 1 and 2 genes have been deleted and replaced by a hormonally active insulin transgene carrying a single amino acid mutation at position B16. These mice are completely protected from T1D and insulitis [23]. Intriguingly, the introduced substitution affects insulin recognition by both CD4+ and CD8+ T cells, as two overlapping immunodominant epitopes have been described in this region: insulin B_9–23_ [24] and insulin B_15–23_ [25], respectively. These data suggest that recognition of these immunodominant epitopes by CD4+ and/or CD8+ T cells may be a mandatory early event in T1D pathogenesis. Studies by Krishnamurthy et al. further corroborated the hypothesis that insulin is the initiating Ag in the T1D of the NOD mouse, because mice rendered tolerant to insulin by transgenic overexpression of insulin 2 in Ag-presenting cells do not develop the immunodominant IGRP_206–214_-specific responses and are protected from T1D [26]. Conversely, mice made tolerant to IGRP by the same means are not protected from T1D [26], suggesting that IGRP-specific responses lay downstream of insulin-specific ones in the pathogenic cascade. The prerequisite of insulin-specific responses for T1D to develop is even found in NOD8.3 mice, which are transgenic for a T-cell receptor recognizing the IGRP_206–214_ epitope [27].

Despite strong evidence pointing to insulin's triggering role, identifying and targeting more *β*-cell Ags remain a high-priority goal. Several considerations justify these efforts. First, the evidence supporting insulin's critical role has been obtained in the NOD model, but similar evidence in human T1D is confined to the early appearance of IAA. Second, once autoimmune T-cell (and B-cell) responses to *β* cells are initiated, the specificity of these responses rapidly enlarges to include more Ags (epitope spreading). As a result, responses to the presumable triggering Ag can be rapidly overgrown by secondary responses, as exemplified by IGRP-specific CD8+ T cells, which rapidly outnumber insulin-specific cells in the NOD model [28]. This consideration is particularly relevant in human, as even prevention trials enroll at-risk subjects at a relatively late stage, once the first signs of *β*-cell autoimmunity (i.e., aAbs) are already detectable, and Ag targeting has probably already diversified. Third, even among inbred NOD mice kept under identical environmental exposure, variations exist in the specificity of CD8+ T-cell responses [29]. Such variations are certainly more extensive in the outbred human population. Fourth, drawing a straight correlation between NOD mice and humans is a gross approximation even in the case of target Ags. Indeed, data in the NOD mouse suggest that GAD and IA-2 are dispensable Ags [30, 31], while they are major targets of aAbs and T-cell responses in humans, and even promising therapeutics in the case of GAD [32]. Of the *β*-cell Ags described, only insulin and GAD have reached the stage of clinical trials and will be discussed, as summarized in Table 1. 

**Figure 2:**
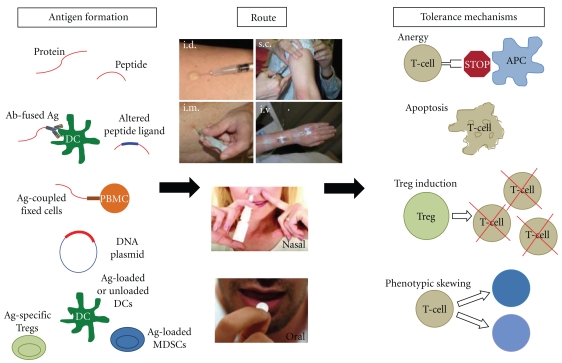
Ag-specific immune therapies. Different Ag formulations can be administered via different routes, triggering various tolerance mechanisms. APC: Ag-presenting cell; DC: dendritic cell; i.d.: intradermal; i.m., intramuscular; i.v.: intravenous; MDSCs: myeloid-derived suppressor cells; PBMC: peripheral blood mononuclear cell; s.c.: subcutaneous; Tregs: regulatory T cells.

Ag-based biologics can be divided into the following categories (Figure 2):

- whole proteins,
- peptides,
- modified protein and peptide Ags,
- DNA plasmids,
- Ag-specific cell therapies.

## 6. Whole Proteins

The main advantage of using whole proteins is that they cover the complete amino acid sequence potentially available for epitope processing and presentation by Ag-presenting cells. Contrary to peptide epitopes, one single agent can be used in all patients, independently of their HLA haplotypes. However, production of the protein in recombinant form at sufficient purity and clinical grade can be challenging, as these preparations are frequently spiked with small contaminants carried over from the host bacteria, yeasts, or baculoviral systems that need to be removed. Only insulin and GAD have so far been employed as whole protein agents for T1D. 

### 6.1. Insulin

The rationale of insulin-based T1D clinical trials is twofold. First, to restore insulin-specific immune tolerance. Second, to put the *β* cell “at rest”, by providing hormonally active exogenous insulin, thus avoiding to overload the endogenous secretory capacity. Indeed, metabolic stress could not only precipitate *β*-cell apoptosis [33], but also make *β* cells more immunogenic and thus susceptible to destruction by autoreactive T cells [34].

Despite this appealing rationale and solid (pre)clinical grounds obtained in the NOD mouse [35–37] and in small-scale pilot human studies [38–40], a number of insulin-based trials, both preventative and interventional, have been disappointingly unsuccessful. Insulin of human origin was used in all of these studies. In the diabetes prevention trial-1 (DPT-1) [41], subjects at high risk of developing T1D were enrolled. These were defined as being positive for islet cell aAbs and already displaying early alterations of insulin secretion, documented by loss of the first-phase insulin response. For such subjects, the projected 5-year risk is >50%. Participants were treated with parenteral insulin—subcutaneous injections twice daily, plus annual intravenous infusions—for a median followup of 3.7 years. Subcutaneous ultralente insulin was administered at a dose of 0.125 U/kg twice daily and intravenous regular insulin given every 12 months for 4 days at a basal rate of 0.015 U/kg/h, which was increased for meals. Despite this intensive treatment, there was no protection on subsequent T1D development. Similar results were obtained in a smaller European trial employing subcutaneous insulin [42]. This outcome is not as surprising when comparing the DPT-1 strategy with its founding preclinical studies in the NOD mice [35]. Indeed, NOD mice were treated at doses of 0.5 U per animal, that is, ~25 U/kg, a dose which is 200-fold higher of what was used in the DPT-1. Regimens like those used in mouse studies would correspond to 1,750 U for an average adult human, which is far above the maximal tolerable dose. Moreover, in most studies, NOD mice were treated continuously for up to 6 months. It is, thus, possible that insulin treated ongoing diabetes rather than preventing incipient disease in some animals. The risk of hypoglycemia is a major concern for translation into human, while mice are quite resistant to insulin-induced hypoglycemia, possibly due to stronger counter-regulatory hormone responses. Longer-acting insulins such as glargine are now available which display a lower risk of hypoglycemia and constant levels throughout most of the day. This pharmacokinetic profile could be interesting not only in widening the therapeutic window of safe dosage, but also by providing low level Ag persistence without significant pulsatility, which may be more effective for tolerance induction [43]. Surprisingly, a mutated insulin (B25Asp) devoid of hypoglycemic activity (due to very low binding affinity for the insulin receptor) and preserving immunogenicity [44] has never been considered for clinical trials, despite encouraging studies in the NOD mouse [45]. B25Asp insulin could be administered at much higher doses, similar to those used in mice, while minimizing the risk of hypoglycemia.

The DPT-1 trial also comprised an oral arm, in which at-risk subjects were treated by oral insulin (7.5 mg/day) for a median of 4.3 years. Also, in this case, no significant protection was induced, in line with the negative results of smaller trials [46–48]. However, analysis of a subgroup positive for IAA suggested a potential benefit, as the annualized T1D rate was 6.2% with oral insulin and 10.4% with placebo [49]. It is possible that the intervention may be more effective in these individuals due to a more active autoimmunity against insulin. This observation has prompted a new oral insulin trial focused on IAA+ at-risk subjects (ClinicalTrials.gov NCT00419562). It also underlines the importance of a through autoimmunity profiling to optimize enrollment (see below). 

 Similar prevention and intervention trials using intranasal insulin administration proved safe but did not yield significant T1D protection [50–52]. One further prevention trial is ongoing (ClinicalTrial.gov NCT00336674). Interestingly, no study has so far tried to administer PI rather than insulin. This could be attractive for a number of reasons. First, PI has a much lower affinity for the insulin receptor. Although it could be degraded into insulin once administered, the window of safe doses not engendering hypoglycemia could be wider. Second, a number of critical epitopes have been described which are specific of PI, as they reside either in the C peptide or at its junction with the B chain [19, 53]. Important epitopes have also been described which are specific of PPI, as they lie in the leader sequence [19, 34, 54]. Although PPI is more difficult to produce due to its lower solubility in water, this characteristic could be even advantageous to obtain a depot effect once injected subcutaneously.

### 6.2. GAD

In a recent intervention trial, Ludvigsson et al. treated new-onset (<18 mo) T1D children positive for anti-GAD aAbs with two subcutaneous injections of GAD (20 *μ*g) or placebo in alum adjuvant and followed this children for 30 months. Although there was no change in insulin requirements, there was a slower decline in the fasting and stimulated C-peptide secretion (a common measure of residual *β*-cell function). Importantly, this effect was observed only in those patients treated within 6 months of diagnosis. Similar observations have been made in other clinical trials, such as the European anti-CD3 trial, where lower insulin requirements were observed only in those patients who had higher residual *β*-cell function at the time of enrollment [55]. Taken together, they suggest that “the earlier the better,” and that interventions at a preclinical stage would be much more beneficial. 

 In the GAD trial, the effect on C-peptide secretion was accompanied by an increase in anti-GAD aAb titers and in mRNA expression of FoxP3 and transforming growth factor (TGF)-*β* in peripheral blood mononuclear cells, which could suggest a regulatory switch. It may be counter-intuitive that the GAD vaccine is administered along with an alum adjuvant, which is known to favor immunogenic rather than tolerogenic responses, especially with the “prime-boost” strategy used in this trial. This peculiarity may raise the possibility that the therapeutic effect may be due, at least in part, to the high titers of anti-GAD Abs induced after vaccination [56]. Indeed, a protective effect of anti-islet aAbs has been evoked to explain the lower T1D risk observed in the offspring of aAb+ T1D mothers compared to aAb-negative ones [57]. Alternative strategies using GAD vaccination in the absence of adjuvant would be equally worth testing. Larger intervention trials using the GAD alum preparation are in progress in Europe and US and will allow to validate the effect on C-peptide secretion and to further explore its immune correlates (ClinicalTrials.gov NCT00723411, NCT00751842, and NCT00529399). A prevention trial is also in progress in Sweden (NCT01122446).

## 7. Peptides

Peptides have the significant edge of being easier to synthesize at high purity than recombinant proteins. Long peptides may have the advantage of covering multiple epitopes, thus targeting different T-cell specificity. Moreover, the use of long peptides requires processing before loading on HLA molecules. This requirement may allow some selectivity in the Ag-presenting cells targeted, as only professional Ag-presenting cells—most notably immature, tolerogenic dendritic cells—can efficiently process and present exogenous peptides. However, there are also multiple drawbacks compared to protein Ags. First, although peptides are smaller molecules and thus deliver up to 50 times more agent on a weight-for-weight basis when compared with protein Ags, the life span of peptides in blood—and likely in other tissues—is very short, in the order of minutes [58]. Second, peptides (but also epitopes processed from protein Ags) may be presented in alternative ways, thus triggering unwanted T-cell activation [59, 60]. Third, the same peptide dose can trigger diverse effects on different T cells, depending on their relative Ag avidity (e.g., simple activation versus activation-induced apoptosis) [61, 62]. Fourth, activation-induced apoptosis, which is one of the mechanisms through which peptides may work, develops through a phase of deleterious activation before driving T cells to death. The drawbacks of this type of peptide-based approaches are exemplified by the disappointing results obtained on multiple sclerosis patients. Two clinical trials were prematurely terminated because of disease flare-ups rather than remissions [63–65]. Although these trials employed altered peptide ligands (see below), the same caveats may apply to native peptides. 

There is no sufficient hindsight from T1D clinical trials to judge whether these theoretical hurdles are encountered. In a Phase I study on 12 new-onset T1D patients, a single intramuscular administration of insulin B chain (i.e., a 30-amino-acid polypeptide) or placebo in incomplete Freund's adjuvant was safe and induced robust insulin-specific humoral and T-cell responses but no difference in stimulated C-peptide responses. Given the small sample size and patients' recruitment irrespective of residual C-peptide secretion, it is not possible to draw conclusions concerning clinical efficacy. However, B-chain-stimulated CD4+ T cells from B-chain-treated patients exhibited higher TGF-*β* secretion in the first 3 months after treatment as compared to T cells from placebo-treated patients, yielding clones with profiles suggestive of a regulatory phenotype [66].

A Phase I clinical trial with a DR*04:01-restricted PI peptide C19-A3 in long-standing (>5 years) T1D patients has also been reported [67]. This trial employed intradermal administration at a dose of 30 or 300 *μ*g repeated 3 times within 2 months, enrolling 18 patients in each arm (peptide versus no treatment). The therapy was well tolerated and did not induce proinflammatory reactivation of PI-specific T cells, but rather transient PI-specific IL-10 secretion in 3 of the 18 patients treated at the lower dose. As is the case for Phase I trials, the aim of this study was to assess safety and not clinical efficacy, and the inclusion criteria reflect this rationale (long-standing T1D patients with glucagon-stimulated C peptide ≤0.2 nmol/L; control group left untreated, i.e., no placebo). Although the observed safety profile is reassuring, it should be noted that PI-specific T-cell responses were not detectable at baseline in these long-standing patients, contrary to what previously observed in new-onset ones [68]. Thus, the effect of peptide administration may be different in the presence of recently *in vivo* primed PI-specific T cells and of a higher residual *β*-cell mass. 

 Clinical trials with a peptide derived from the heat-shock protein (hsp60) immunodominant epitope 437–460 (DiaPep277) showed marginally preserved endogenous insulin production in some newly diagnosed T1D patients [69, 70]. The mechanism by which the DiaPep277 may function is not completely understood. Qualifying hsp60 as a target Ag in T1D autoimmunity is debatable, and alternative, non-Ag-specific mechanisms are possible. Indeed, hsp60 has been shown to activate T cells via the Toll-like receptor 2 and to inhibit T-cell chemotaxis [71]. This pathway could also induce the shift from T helper (Th)1 to Th2 cytokines observed in humans. Thus, this mode of action would categorize DiaPep277 as a systemic rather than an Ag-specific immune modulator.

## 8. Modified Protein and Peptide Ags

Disarming pathogenic T cells by modifying the epitopes they recognize at key amino acid positions is an option that has been sought in several autoimmune diseases. TCR interaction with these modified epitopes, called altered peptide ligands (APLs), can result in only partial activation and dramatically different T-cell phenotypes, ranging from inducing selective stimulatory functions to completely turning off their functional capacity [72]. The central role of the insulin B_9–23_ epitope in the NOD mouse [23, 73] and its immunodominance in T1D patients [74] led to develop an APL version called NBI-6024. This B_9–23_ APL showed promising results in NOD mice [75]. Results of a Phase I clinical study on recent-onset T1D patients suggested that NBI-6024 treatment shifted Th1 responses to a Th2 phenotype [76]. However, a subsequent Phase II, dose-ranging trial testing repeated NBI-6024 subcutaneous treatment at 0.1, 0.5, or 1 mg did not preserve *β*-cell function [77]. Clinical trials with APLs in multiple sclerosis further chilled down enthusiasm for this approach. Two Phase II trials with a myelin basic protein APL were prematurely terminated, as treatment led to disease exacerbations and hypersensitivity reactions [63, 64]. This dramatic experience underlines the difficulty of developing one single APL which would induce the same effect on all cognate T cells. It is possible that the APL effect may be dependent on parameters such as effective dose delivered and T-cell avidity, activation status and phenotype, and thus trigger different polarizing signals [65].

As DCs present Ags in a tolerogenic manner in the steady state, for example, in the absence of infection or inflammation, selective Ag delivery to DCs promotes tolerance in the absence of maturation stimuli [78–80]. To this end, a number of mAbs against endocytic receptors expressed by DCs have been developed, with the aim of using them to target Ag delivery. DEC-205 (CD205) is one of such receptors, which is expressed on the CD8+ DC subset capable to efficiently cross-present [81]. Besides selective targeting, this *in vivo* Ag delivery approach would obviate the need for leukapheresis and *ex vivo* DC manipulation. On these grounds, the group of T. DiLorenzo delivered the IGRP mimotope NRP-V7 *in vivo* to murine DCs by fusing it with an anti-DEC-205 mAb. Proliferation of transferred NRP-V7-specific T cells was initially observed, but this was followed by deletion. Tolerance was achieved because rechallenge of mice with NRP-V7 in adjuvant did not induce an immune response. NRP-V7 delivery through DEC-205 was tolerogenic only when performed in the steady state. As expected, co-administration of anti-CD40 mAb and polyI:C to mature DCs led instead to enhanced expansion of transferred T cells [82]. Although evidence of T1D protection is still missing, this approach may prove of great interest when combined with suitable epitopes. Moving towards this direction, the feasibility of DEC-205 delivery of the entire PPI molecule is under investigation [83]. This would facilitate translation to patients expressing diverse MHC molecules. 

 Another original system of tolerogenic Ag delivery has been developed by Miller and coworkers [84–86]. This consists in administration of self peptides covalently cross-linked to cells via ethylene carbodiimide (ECDI). In preclinical models of various autoimmune diseases, this approach involves ECDI cross-linking of self proteins or peptides to syngeneic splenocytes. Intravenous injection of these Ag-coupled splenocytes is highly effective at inducing tolerance for both prevention and treatment of various autoimmune diseases in mouse models, including experimental autoimmune encephalomyelitis (a mouse model of multiple sclerosis) [87] and T1D in the NOD mouse [88]. Ag-coupled splenocytes behave like apoptotic bodies and are rapidly uptaken by Ag-presenting cells, much more efficiently than soluble protein or peptide Ags [89]. Ag presentation leads to tolerance induction by inducing Ag-specific T-cell unresponsiveness *via* two synergistic mechanisms: programmed death (PD)-1/PD ligand 1-mediated anergy and T regulatory cell (Treg) activation [88]. Processing and presentation of Ags coupled to apoptotic bodies gives the advantage of inducing tolerance by cellular carriers fixed with peptides, intact proteins, or even crude homogenates of the target organ [87]. This tolerogenic system is currently being tested in multiple sclerosis, using ECDI-fixed autologous peripheral blood leukocytes coupled with a cocktail of seven myelin peptides. A similar trial using insulin-coupled autologous leukocytes for prevention of T1D is under development by the Immune Tolerance Network.

## 9. DNA Plasmids

DNA vaccines are the simplest embodiment of vaccines that, rather than consisting of the Ag itself, provide genes for endogenous synthesis of the protein Ag. The idea stemmed from the need to efficiently deliver protein and polypeptide Ags to the MHC Class I pathway for presentation to and stimulation of CD8+ T-cell responses. Inducing such responses with exogenous protein Ags is not efficient, as it requires cross-presentation, a requirement which can be fulfilled mostly if not exclusively by specialized DC subsets. As DNA-encoded Ags are endogenously translated by transfected cells, DNA immunization has the potential to result in conventional priming as well as cross-priming. 

 There are many ways to efficiently deliver DNA vaccines [90], and this strategy has been exploited for many research applications such as T-cell epitope identification [91, 92]. One strategy consists of bombarding the epidermis with gold microbeads coated with plasmid, which can directly transfect Langerhans cells causing their rapid migration to draining lymph nodes. Alternatively, plasmids can be intramuscularly injected, leading to predominant transfection of myocytes and less efficient Ag presentation. This latter approach is currently been explored for tolerance induction, using a plasmid encoding for proinsulin (BHT-3021) in T1D patients with less than 5 years of disease (ClinicalTrials.gov NCT00453375). The relatively long disease duration used for patient recruitment and the low potency of DNA vaccines documented in other clinical trials may be important factors in the final outcome. 

## 10. Ag-Specific Cell Therapies

Ag-specific tolerance restoration could be induced not only by using suitable Ag proteins or peptides, but also by adoptively transferring cells armed to be selective for the targets of choice. These approaches are being explored for Tregs and DCs [93].

Expansion protocols to obtain high numbers of CD4+ Tregs suitable for *in vivo* cell therapies have been developed for both mouse and human [94–97]. However, these cells remain polyclonal in nature although a shortcut has been obtained in murine models by expanding T cells from mice transgenic for a T-cell receptor which recognizes a specific *β*-cell epitope such as BDC2.5. Although some pioneering reports of expanded human *β*-cell Ag-specific Tregs have been published [98, 99], it is not clear whether these cells would have suitable characteristics to exert therapeutic effects once transferred *in vivo*. Further incertitude is added by a recent paper describing that polyclonal murine CD4+ Tregs can revert their phenotype after *in vivo* transfer, losing FoxP3 expression and acquiring pathogenic potential. Indeed, these “ex-Tregs” were found to accumulate spontaneously in the insulitis of NOD mice, suggesting that this phenomenon may not be peculiar of transfer settings. Even more worryingly, these ex-Tregs were capable of efficiently transferring disease, similar to standard T effector cells [100].

Another cell-based approach consists of loading immature DCs with the Ag of interest. This type of approach has previously been shown to be efficient at promoting Ag-specific immune tolerance [101, 102]. This strategy is now being tested for T1D intervention in a Phase I safety study led by M. Trucco. Tolerogenic autologous monocyte-derived DCs are being generated by treating them *ex vivo* with antisense oligonucleotides targeting the CD40, CD80, and CD86 costimulatory molecules [103]. These modified DCs are being compared with control autologous monocyte-derived DCs left untreated. The fact that intravenously administered bone-marrow-derived DCs accumulate predominantly in the spleen and the pancreatic and tracheal lymph nodes [104] justifies the choice of not loading these DCs with exogenous Ag. These DCs may acquire islet Ags *in vivo *in pancreatic lymph nodes and could thus modulate effector and regulatory T-cell responses to T1D-relevant Ags even without deliberate prior Ag loading. The other peculiarity of this trial is that DCs (which are not used fresh but cryopreserved) will be administered intradermally closest to the physical location of the pancreas, as both sites may drain to the same lymph nodes (ClinicalTrials.gov NCT00445913). Importantly, preclinical studies suggest that the therapeutic effect of these unloaded tolerogenic DCs remains Ag-specific, as splenic T cells from treated mice proliferated to allo-Ags *ex vivo* [103]. 

 Another cell population which may soon be tested for cell-based immunotherapy is myeloid-derived suppressor cells (MDSCs). These are cells of myeloid origin with immunoregulatory activity that can suppress Ag-specific and nonspecific T-cell responses *via* different mechanisms in cancer [105–107], transplantation [108], and T1D [109]. Pre-clinical study testing the protective role of these cells in T1D were performed in RIP-HA mice transferred with HA-specific CD4+ T cells, where it was shown that HA-loaded MDSCs could act as APCs in an Ag-specific fashion to induce anergy of effector T cells, development of Tregs, and T1D prevention [109].

## 11. The Importance of Immune Surrogate Markers

**Figure 3:**
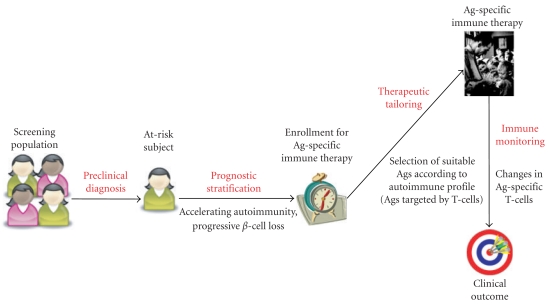
“Immune staging" of T1D. Biomarkers of *β*-cell autoimmunity such as aAbs and T cells could help to identify at-risk subjects at an early stage (preclinical diagnosis) and to follow them up over time to decide the need for immune therapy and the best timing for treatment (prognostic stratification). Ag-specific immune therapy could be personalized for each subject by administering therapeutic formulations of those Ags targeted by aAb and/or T-cell responses. Modifications induced on such responses could be followed in real time during treatment, thus allowing to assess immune efficacy prior to and independent of clinical outcome.

In light of the formidable challenge of rescuing a significant *β*-cell mass in already diabetic patients, it is important to evaluate not only clinical (lower HbA1c values and reduced insulin requirements) and metabolic (C-peptide secretion) endpoints, but also immune surrogate endpoints. This is even more important in trials not showing clinical benefit, or not poised to detect such benefits, as is commonly the case for phase I studies. Indeed, the lack of clinical effects could be open to disparate interpretation, including late treatment in front of full-blown disease, the need to target multiple Ags in advanced T1D once epitope spreading has occurred, or failure to restore immune tolerance. These possibilities can be sorted out by immune monitoring analyses performed before, during, and after treatment, particularly by scrutinizing changes induced in T-cell responses specific to the administered Ag (Figure 3). Validation and standardization of blood sample processing and T-cell assay procedures is an important goal to this end [110–112]. 

 We recently reported a proof-of-concept study on aAb+ diabetic patients not requiring insulin at the time of diagnosis. These patients were treated with intranasal insulin in an attempt to save residual *β* cells. Although nasal insulin treated patients eventually progressed towards insulin dependency at a rate similar to placebo-treated ones, we could document successful induction of insulin-specific immune tolerance both at the T-cell and antibody level [52]. Contrary to what observed in the placebo arm, patients treated with intranasal insulin displayed significant reductions in frequencies of interferon-*γ*-secreting PI-specific T-cell responses, using high sensitivity T-cell assays that amplify responses by means of an accelerated cocultured DC (acDC) stimulation (Martinuzzi et al., under revision). This effect was Ag-specific, as it was not observed for responses towards the tetanus toxoid recall Ag. The initiation of insulin therapy further documented that this PI-specific tolerance was operational *in vivo*, as intranasal insulin treated subjects failed to develop anti-insulin antibodies [52]. These results suggest that this intervention is immunological effective, but not sufficient to rescue *β* cells, probably because administered too late, at an advanced stage where most islets have already disappeared and *β*-cell autoimmunity has already spread too far on additional Ag specificities. Knowing whether Ag-specific T-cell responses are modified and in which individuals provides key mechanistic information to plan further trials and modify therapeutic strategies accordingly. This information may also be critical to optimize enrollment strategies. Indeed, pretreatment testing for T-cell reactivities could allow to focus enrollment on those patients displaying active insulin-specific autoimmune responses, as there might be little benefit in treating those who do not harbor such responses (Figure 3). Imaging techniques allowing to visualize *β*-cell mass and infiltration will provide additional tools for immune-based pretreatment staging and post-treatment monitoring [113].

## 12. Combination Therapies

Despite the mentioned evidence in the NOD mouse for an initiating role of insulin as the primary *β*-cell Ag target, the same principle may not apply to outbred humans, due to the variable genetic and environmental background and the later timing of intervention and even prevention trials, at a stage where epitope spreading is likely to have occurred. Therefore, strategies where multiple *β*-cell Ags (e.g., PI and GAD) are combined may yield synergistic effects and more substantial clinical benefit. This possibility will certainly be explored in upcoming clinical trials. Another option under consideration consists of combining Ags with immune modulatory mAbs such as anti-CD3 and anti-CD20, which have already been trialed alone, sometimes with encouraging results [114]. Besides the potential for synergy, this approach may allow to reduce mAb dosing, thus limiting unwanted side effects and facilitating repeated treatment [115–117]. Combination of tolerogenic strategies with approaches aimed at replacing [118, 119] or regenerating [120] *β* cells are also attractive, as they may allow to intervene at later stages. Although a first clinical trial testing exenatide alone or in combination with daclizumab did not show any effect [121], other agents and combinations thereof need to be tested before drawing definite conclusions.

## 13. Conclusions

We are moving towards a new era of clinical trials in T1D, where the long-sought goal of Ag-specific immune tolerance is becoming at reach. To turn these promises into reality, it will be important to comparatively evaluate several aspects that remain poorly defined. (1) Administration routes: intravenous, subcutaneous, intradermal, and mucosal. Regarding the latter, it is surprising that only oral and intranasal routes have been investigated for T1D, leaving out the sublingual approach despite its successful track record for allergy desensitization [122]. (2) Ag dose, identifying suitable therapeutic ranges, which will critically depend on the nature of the Ag and the route of administration. We have discussed the hurdles of translating the insulin doses used in NOD mouse studies into clinical trials. Importantly, difference insulin formulations may widen the therapeutic window. Another key difference between preclinical and human studies is the timing of intervention. A proposed primary prevention trial (Pre-POINT) [123] aims to address this issue of disease stage by intervening with oral or nasal insulin in children genetically predisposed to T1D, before the appearance of the first signs of *β*-cell autoimmunity (i.e., in children who have not yet developed IAA). (3) Frequency and interval of administration, keeping in mind that approaches promoting longer persistence of low Ag loads may be beneficial over high-load spikes [43]. Ancillary mechanistic studies should accompany these trials to clarify modes of action and help optimizing the risk-to-benefit ratio. Autoimmune T-cell profiling may be particularly useful for tailoring and monitoring treatment in each individual patient.

## Figures and Tables

**Table 1:** Clinical trials in T1D using antigen-specific strategies.

Antigen type	Antigen	Formulation	Route	Trial (Phase)	Subjects	Outcome and/or immune biomarkers	Reference
Protein	Insulin	Short-acting insulin	Intravenous	Intravenous insulin (Phase I)	Recent-onset T1D	Higher stimulated C peptide and lower HbA1c versus s.c. NPH insulin	[38]
Protein	Insulin	(Ultra)lente/regular insulin (s.c.) + short-acting insulin (i.v.)	Subcutaneous + Intravenous	Joslin Insulin Prophylaxis Trial (Phase I)	At risk	Suggestive of efficacy	[39]
Protein	Insulin	Lente/short-acting insulin (s.c.) + short-acting insulin (i.v.)	Subcutaneous + Intravenous	Schwabing Insulin Prophylaxis Trial (Phase I)	At risk	Suggestive of efficacy	[40]
Protein	Insulin	Ultralente insulin (s.c.) + short-acting insulin (i.v.)	Subcutaneous + Intravenous	DPT-1 Parenteral arm (Phase III)	At risk	No effect	[41]
Protein	Insulin	Ultralente insulin	Subcutaneous	EPPSCIT (Phase II)	At risk	No effect	[42]
Protein	Insulin	Short-acting insulin	Oral	ORALE (Phase II)	Recent-onset T1D	No effect	[46]
Protein	Insulin	Short-acting insulin	Oral	IMDIAB VII (Phase II)	Recent-onset T1D	No effect	[47]
Protein	Insulin	Short-acting insulin	Oral	DPT-1 Oral arm (Phase III)	At risk	Some efficacy in IAA^+^ subjects	[49]
Protein	Insulin	Short-acting insulin	Oral	Oral insulin tolerance (Phase III)	Recent-onset T1D	1 mg improved C-peptide responses in older patients;10 mg accelerated C-peptide decline in younger patients	[48]
Protein	Insulin	Short-acting insulin	Oral	NIH/ADA/JDRF oral insulin (Phase III)	At-risk IAA^+^	Ongoing	NCT 00419562
Protein	Insulin	Short-acting insulin	Intranasal	INIT-I (Phase I)	At-risk IAA^+^	Increase in aAb and decrease in T-cell proliferative responses to insulin	[50]
Protein	Insulin	Short-acting insulin	Intranasal	DIPP (Phase III)	At risk	No effect	[51]
Protein	Insulin	Short-acting insulin	Intranasal	Intranasal insulin in T1D patients (Phase II)	Recent-onset, non-insulin-dependent T1D	No effect; decrease in IFN-*γ* T-cell responses to PI; decrease in Ab responses to exogenous insulin	[52]
Protein	Insulin	Short-acting insulin	Intranasal	INIT-II (Phase II)	At risk with preserved 1st phase insulin response	Ongoing	NCT 00336674
Protein	Insulin	Short-acting insulin	Oral or Intranasal	Pre-POINT	IAA^−^ chidren at high genetic risk for T1D	Planned	[123]
Protein	GAD	Recombinant GAD in alum (Diamyd)	Subcutaneous	Swedish Diamyd (Phase II)	Recent-onset T1D	Slower decline in fasting and stimulated C-peptide secretion; increase in anti-GAD aAbs and in FowP3 and TGF-*β* mRNA	[32]
Protein	GAD	Recombinant GAD in alum (Diamyd)	Subcutaneous	EU Diamyd (Phase III)	Recent-onset T1D	Ongoing	NCT 00723411
Protein	GAD	Recombinant GAD in alum (Diamyd)	Subcutaneous	US Diamyd (DIAPREVENT) (Phase III)	Recent-onset T1D	Ongoing	NCT 00751842
Protein	GAD	Recombinant GAD in alum	Subcutaneous	NIDDK/ADA/JDRF GAD-alum (Phase II)	Recent-onset T1D	Ongoing	NCT 00529399
Protein	GAD	Recombinant GAD in alum (Diamyd)	Subcutaneous	DIAPREV-IT (Phase II)	At risk, GAD aAb^+^+ ≥1 other aAb	Ongoing	NCT 01122446
Peptide	Insulin	Insulin B chain in incomplete Freund's adjuvant	Intramuscular	IBC-VS01 (Phase I)	Recent-onset IAA^+^ T1D	Increased TGF-*β* production	[66]
Peptide	Proinsulin	PI_C19- A3_	Intradermal	PI peptide immunotherapy (Phase I)	Long-standing T1D	Transient PI-specific IL-10 secretion in 3/18 patients at 30 *μ*g	[67]
Modified peptide	Insulin	NBI-6024 (B_9–23_ APL)	Subcutaneous	NBI-6024-0003 (Phase I)	Recent-onset T1D	Shift from Th1 to Th2 responses	[76]
Modified peptide	Insulin	NBI-6024 (B_9–23_ APL)	Subcutaneous	Neurocrine NBI-6024 (Phase II)	Recent-onset T1D	No effect	[77]
Modified protein	Insulin	Insulin-coupled ECDI-fixed autologous leukocytes	?	ITN insulin-coupled leukocytes	At risk	Planned	[86]
DNA plasmid	Proinsulin	BHT-3021 (PI plasmid)	Intramuscular	Bayhill BHT-3021 (Phase I)	Recent-onset T1D	Ongoing	NCT 00453375
Ag-specific cell therapy	None	Autologous monocyte-derived DCs treated with CD40/CD80/CD86 antisense oligonucleotides	Intradermal	Pittsburgh DC vaccine (Phase I)	Long-standing T1D	Ongoing	NCT 00445913
